# Hypoxanthine-Guanine Phosphoribosyltransferase/adenylate Kinase From *Zobellia galactanivorans*: A Bifunctional Catalyst for the Synthesis of Nucleoside-5′-Mono-, Di- and Triphosphates

**DOI:** 10.3389/fbioe.2020.00677

**Published:** 2020-06-24

**Authors:** Javier Acosta, Jon Del Arco, Maria Luisa Del Pozo, Beliña Herrera-Tapias, Vicente Javier Clemente-Suárez, José Berenguer, Aurelio Hidalgo, Jesús Fernández-Lucas

**Affiliations:** ^1^Applied Biotechnology Group, Universidad Europea de Madrid, Urbanización El Bosque, Madrid, Spain; ^2^Centro de Biología Molecular Severo Ochoa (CSIC-UAM), Madrid, Spain; ^3^Grupo de Investigación en Ciencias Naturales y Exactas, GICNEX, Universidad de la Costa, CUC, Barranquilla, Colombia; ^4^Faculty of Sport Sciences, Universidad Europea de Madrid, Urbanización El Bosque, Madrid, Spain

**Keywords:** enzymatic synthesis, nucleotides, phosphoribosyltransferase, nucleoside-5cpsdummy′-monophosphate kinase, dual domain protein

## Abstract

In our search for novel biocatalysts for the synthesis of nucleic acid derivatives, we found a good candidate in a putative dual-domain hypoxanthine-guanine phosphoribosyltransferase (HGPRT)/adenylate kinase (AMPK) from *Zobellia galactanivorans* (*Zg*HGPRT/AMPK). In this respect, we report for the first time the recombinant expression, production, and characterization of a bifunctional HGPRT/AMPK. Biochemical characterization of the recombinant protein indicates that the enzyme is a homodimer, with high activity in the pH range 6-7 and in a temperature interval from 30 to 80°C. Thermal denaturation experiments revealed that *Zg*HGPRT/AMPK exhibits an apparent unfolding temperature (*Tm*) of 45°C and a retained activity of around 80% when incubated at 40°C for 240 min. This bifunctional enzyme shows a dependence on divalent cations, with a remarkable preference for Mg^2+^ and Co^2+^ as cofactors. More interestingly, substrate specificity studies revealed *Zg*HGPRT/AMPK as a bifunctional enzyme, which acts as phosphoribosyltransferase or adenylate kinase depending upon the nature of the substrate. Finally, to assess the potential of *Zg*HGPRT/AMPK as biocatalyst for the synthesis of nucleoside-5′-mono, di- and triphosphates, the kinetic analysis of both activities (phosphoribosyltransferase and adenylate kinase) and the effect of water-miscible solvents on enzyme activity were studied.

## Introduction

Purine nucleotides are involved in multitude of biochemical processes, but they are also particularly important as building blocks for RNA and DNA synthesis. Biosynthesis of purine nucleotides is performed through two different metabolic routes, *de novo* and salvage pathways. In the *de novo* pathway, purine nucleotides are synthesized from simple precursors like glycine, glutamine, or aspartate. In contrast, salvage pathway employs purine nucleobases to generate the corresponding nucleoside-5′-monophosphates (NMPs). This requirement for purines is satisfied by means of different endogenous and/or exogenous sources of preformed nitrogen bases (el Kouni, 2003; Del Arco and Fernández-Lucas, 2018). Both metabolic routes, *de novo* and salvage pathways, lead to inosine-5′-monophosphate (IMP) synthesis. IMP is converted to guanosine-5′-monophosphate (GMP) and adenosine-5′-monophosphate (AMP), which are subsequently phosphorylated to get guanosine-5′-triphosphate (GTP) and adenosine-5′-triphosphate (ATP), respectively.

Biocatalysis aims to reproduce, implement and expand nature’s synthetic strategies to perform the synthesis of different organic compounds using whole cells or enzymes.

The chemical synthesis of nucleotides proceeds through the mono-, di-or triphosphorylation of precursor nucleosides. However, chemical methodologies require the use of chemical reagents (phosphoryl chloride, POCl_3_, or phosphorus pentoxide, P_2_O_5_), acidic conditions, and organic solvents, which are expensive and environmentally harmful (Yoshikawa et al., 1967, 1969). In addition, chemical synthesis of the precursor nucleosides requires the protection and de-protection of functional groups, as well as the isolation of intermediates. It leads to poor or moderate global yields and low product purity, and therefore an increase in production costs. In contrast, enzymatic bioprocesses offers many different advantages, such as the possibility of one-pot reactions under mild conditions, high chemo-, regio- and stereoselectivity, and an eco-friendly technology (Del Arco and Fernández-Lucas, 2017; Acosta et al., 2018; Del Arco et al., 2018b).

In this sense, the use of enzymes from purine and pyrimidine salvage pathway as biocatalysts for the synthesis of nucleosides and nucleotides has been extensively reported (Mikhailopulo, 2007; Fernández-Lucas et al., 2010; Fresco-Taboada et al., 2013; Fernández-Lucas, 2015; Lapponi et al., 2016; Del Arco and Fernández-Lucas, 2017; Ding and Ou, 2017; Lewkowicz and Iribarren, 2017; Serra et al., 2017; Acosta et al., 2018; Del Arco et al., 2018b, 2019a; Pérez et al., 2018; Kamel et al., 2019; Ubiali and Speranza, 2019).

**FIGURE 1:**
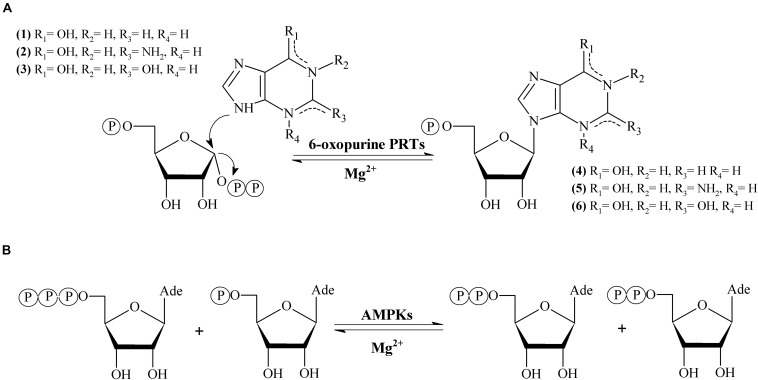
**(A)** Enzymatic synthesis of 6-oxopurine purine nucleosides monophosphate catalyzed by 6-oxopurine PRTs. **(B)** Enzymatic synthesis adenine nucleotides catalyzed by AMPKs.

6-oxopurine phosphoribosyltransferases (6-oxo PRTs, EC 2.4.2.8, EC 2.4.2.22) are essential enzymes in the purine salvage pathway (el Kouni, 2003). 6-oxo PRTs catalyze the reversible transfer of the 5-phosphoribosyl group from 5-phospho-α-D-ribosyl-1-pyrophosphate (PRPP) to N9 on the 6-oxopurine bases hypoxanthine (1), guanine (2) or xanthine (3) to form IMP (4), GMP (5) or XMP (6) (HPRT, GPRT, XPRT, HGPRT, GXPRT or HGXPRT), respectively, in the presence of Mg^2+^ (Del Arco et al., 2017, 2018c; Figure 1A).

Adenylate kinase (EC 2.7.4.3, AMPK) belongs to nucleoside-5′-monophosphate kinase (NMPK) family. AMPK catalyzes the reversible transfer of the terminal phosphate group between ATP and AMP to give 2 molecules of ADP in presence of Mg^2+^ (Figure 1B). AMPK is present in *de novo* synthesis of nucleotides and also plays an essential role in the maintenance of cellular homeostasis of adenine nucleotides by the interconversion of AMP, ADP and ATP (Davlieva and Shamoo, 2010; Panayiotou et al., 2014).

**FIGURE 2:**
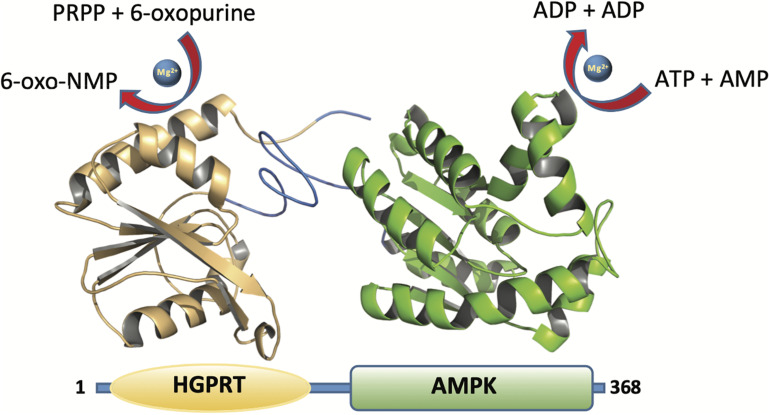
Cartoon representation of the *Zg*HGPRT/AMPK monomer model comprising HGPRT (yellow) and AMPK (green) domains. The figure was prepared with PyMOL (Delano, 2002).

Herein we report, for the first time, a bifunctional protein from *Zobellia galactanivorans* which contains both HGPRT (N-terminal part) and AMPK (C-terminal part) domains (Figure 2). In the N-terminal part a HGPRT module, involved in the purine salvage, converts Hyp to IMP and Gua to GMP. Also, in the C-terminal part an AMPK module, involved in the energy metabolism and nucleotide synthesis, catalyzes the reversible transfer of the terminal phosphate group from ATP to AMP. The recombinant protein (named *Zg*HGPRT/AMPK) was shown as a homodimer, active in the pH interval 6-7 and in a broad temperature range (30–80°C). Moreover, *Zg*HGPRT/AMPK displays an apparent unfolding temperature (*Tm*) of 45°C. Finally, to assess the potential of *Zg*HGPRT/AMPK the kinetic analysis of both activities (phosphoribosyltransferase and adenylate kinase) and the effect of divalent cations and water-miscible solvents on enzyme activity were assayed.

## Materials and Methods

### Materials

Cell culture medium reagents were purchased from Difco (St. Louis, MO, United States). Triethyl ammonium acetate buffer was provided by Sigma-Aldrich (Madrid, Spain). All other reagents and organic solvents were purchased from Symta (Madrid, Spain). Nucleosides, nucleotides and nucleobases were provided by Carbosynth Ltd. (Compton, United Kingdom).

### Cloning, Expression and Protein Purification

After an *in silico* mining of phosphoribosyltransferase (PRT) candidates, we found a gene which encodes a putative bifunctional protein HGPRT/AMPK in *Zobellia galactanivorans* genome (European Nucleotide Archive code: CAZ96627; UniProtKB code G0LC40). The corresponding *hgprt/ampk* gene was provided by GenScript (United States) as a *Nhe*I-*Bam*HI fragment subcloned into the expression vector pET28b (+). The recombinant vector pET28b*_*Zg*_*_H__G__PRT/AMPK_ encodes an N-terminal His_6_-tagged fusion with a thrombin cleavage site between the tag and the enzyme. *Zg*HGPRT/AMPK was expressed in *Escherichia coli* BL21(DE3) grown at 37°C in Luria Bertani medium supplemented with kanamycin (50 μg/mL). Protein overexpression was induced by adding 0.5 mM isopropyl β-D-1-thiogalactopyranoside (IPTG) to exponential cultures and the cells were further grown for 4 h. Then, cells were harvested by centrifugation at 3,800 × *g* and the resulting pellet was resuspended in 10 mM sodium phosphate buffer pH 7. Crude extracts were prepared by cellular disruption of cell suspensions using a digital sonicator. The lysates were centrifuged at 16,500 × *g* for 30 min at 4°C. The cleared lysates were loaded onto a 5-mL HisTrap FF column (GE Healthcare), pre-equilibrated in a binding buffer (20 mM Tris–HCl buffer, pH 7.5, with 100 mM NaCl and 20 mM imidazole). Bound proteins were eluted using a linear gradient of imidazole (from 20 to 500 mM). Fractions containing *Zg*HGPRT/AMPK were identified by SDS-PAGE, pooled, concentrated and loaded onto a HiLoad 16/60 Superdex 200 prep grade column (GE Healthcare) pre-equilibrated in 20 mM sodium phosphate, pH 7. Fractions with the protein of interest were identified by SDS-PAGE. Protein concentration was determined spectrophotometrically by UV absorption 280 nm using a ε_280_ = 22,350 M^–1^cm^–1^.

### Analytical Ultracentrifugation Analysis

Sedimentation velocity experiments for *Zg*HGPRT/AMPK were carried out in 20 mM Tris–HCl (pH 8, 20°C, 50,000 × *g*) using an Optima XL-I analytical ultracentrifuge (Beckman-Coulter Inc.) equipped with UV-VIS absorbance and Raleigh interference detection systems, an An-60Ti rotor and standard (12 mm optical path) double-sector center pieces of Epon-charcoal. Sedimentation profiles were recorded at 292 nm. Sedimentation coefficient distributions were calculated by least-squares boundary modeling of sedimentation velocity using the continuous distribution *c*(*s*) Lamm equation model as implemented by SEDFIT 14.7 g.

Baseline offsets were measured afterward at 200,000 × *g*. The apparent sedimentation coefficient distribution, *c*(*s*), and sedimentation coefficients were calculated from the sedimentation velocity data using SEDFIT (Brown and Schuck, 2006). The experimental sedimentation coefficients were corrected to standard conditions (water, 20°C, and infinite dilution) using SEDNTERP software to obtain the corresponding standard values (*s*_20_,*_w_*) (Laue et al., 1992). The corresponding apparent weight-average molar masses (*M*_w_) were determined from the buoyant masses, considering the partial specific volumes of the protein (0.4 mg/ml) obtained from the amino acid composition using the program SEDNTERP (Minton, 1997).

### Phosphoribosyltransferase Activity Assay

The standard phosphoribosyltransferase activity assay was performed by incubating 10–50 μL of free extracts or 6.5–13 μg of purified enzyme with a 40 μL solution containing 2 mM PRPP, 2 mM Hyp, 2.4 mM MgCl_2_ in 50 mM Tris–HCl pH 8 at 50°C and 300 r.p.m. for 5–10 min After this, the enzyme was inactivated as previously described (Del Arco et al., 2018a) and the IMP production was analyzed and quantitatively measured using HPLC. All determinations were carried out in triplicate and the maximum error was ≤2%. Under such conditions, one activity unit, U (μmol/min), was defined as the amount of enzyme (mg) producing 1 μmol/min of IMP under the assay conditions.

### Influence of pH and Temperature on *Zg*HGPRT/AMPK Activity

The optimum pH of the enzyme was determined under standard phosphoribosyltransferase assay condition, using sodium citrate (pH 4–6), sodium phosphate (pH 6–8.5), MES (pH 5.5–7), Tris–HCl (pH 7–9) and sodium borate (pH 8–11) as reaction buffers (50 mM). The optimum temperature was determined using the standard assay over a 20–80°C range.

### Influence of Divalent Cations on Enzyme Activity

To determine the effect of divalent cations on *Zg*HGPRT/AMPK activity, different divalent salts (MgSO_4_, MnSO_4_, ZnSO_4_, CoSO_4_, and CaCl_2_) were added to the reaction mixture at different concentrations (2–20 mM). The reaction was performed using the standard phosphoribosyltransferase activity assay under the optimal pH and temperature conditions previously determined. In this respect, 6.5 μg of purified enzyme were incubated in a 40 μL solution containing with 2 mM PRPP, 2 mM Hyp and 2–20 mM divalent salts, in 50 mM sodium phosphate buffer pH 7 at 50°C and 300 r.p.m., 5–10 min.

### Influence of Water-Miscible Solvents on Enzyme Activity

To determine the effect of organic solvents on enzymatic activity, the phosphoribosyltransferase activity was assayed in the presence of different protic and aprotic organic solvents. To this end, 6.5 μg of purified enzyme were added to a 40 μL solution containing 2 mM PRPP, 2 mM Hyp, 2.4 mM MgCl_2_ in 50 mM sodium phosphate buffer pH 7, in the presence of 20% (v/v) water-miscible organic solvents. The reaction mixture was incubated at 50°C for 5–10 min (300 r.p.m.).

### Thermal Stability

*Zg*HGPRT/AMPK was stored at 4 and −80°C in 20 mM sodium phosphate buffer, pH 7 for 365 days. Periodically, samples were taken and the enzymatic activity was evaluated. Storage stability was defined as the relative activity between the first and the successive reactions. Moreover, thermal stability was studied by incubating 6.5 μg of purified enzyme in 20 mM sodium phosphate buffer, pH 7, for 240 min at different temperatures (40–60°C).

### Thermal Denaturation

The melting temperature (*T*_m_) was measured using differential scanning fluorimetry in a Rotor Gene^TM^ 6000 (Corbett Life Sciences) essentially as previously described (Niesen et al., 2007; Del Arco et al., 2019b). For this purpose, *Zg*HGPRT/AMPK was diluted to a final concentration of 20 and 18 μL were mixed with 2 μL 100× diluted SYPRO Orange (Sigma-Aldrich, St. Louis, MO, United States). The samples were heated over 35–95°C range at 1°C/min. The fluorescent signal was measured with excitation and emission filters of 460 and 510 nm, respectively. The *T*_m_ was approximated to the melt peak obtained from a graph plotting the negative first derivative of the melting curve with respect to the temperature (dRFU/dT vs. T), where RFU stands for relative fluorescence units.

### Substrate Specificity

To assess the potential of *Zg*HGPRT/AMPK as a bifunctional catalyst, both phosphoribosyltransferase and adenilate kinase activities were assayed.

On the one hand the phosphoribosyltransferase activity of *Zg*HGPRT/AMPK was tested against different purine and pyrimidine nucleobases at different conditions. In this respect, 50 mM sodium phosphate buffer pH 7 was used as reaction buffer when hypoxanthine, adenine, cytosine, uracil or thymine were used as substrates, whereas 50 mM Tris–HCl buffer pH 8 was used as reaction buffer for guanine and xanthine.

One the other hand, the interconversion of adenine nucleotides (ATP, ADP, and AMP) was assayed to evaluate adenylate kinase activity. To this end, 13 μg of purified enzyme were added to a 40 μL solution containing 3.2 mM ATP and 3.2 mM AMP (or 6.4 mM ADP), 12 mM MgCl_2_ in 50 mM sodium phosphate buffer pH 7. The reaction mixture was incubated at 50°C and 300 r.p.m. for 10–20 min.

### Kinetic Analysis

The steady-state kinetic parameters, *K*_M_, *k*_cat_, and *k_cat_/K_M_*, for both HGPRTase and AMPKase activities were determined. The kinetic analysis of HGPRT was performed at varying concentrations of one substrate (0.5–17.2 mM for Hyp, 0.5–17.2 mM for PRPP), fixing the concentration of the other substrate at constant saturating level (5 mM). An identical approach was followed for the kinetic analysis of AMPKase activity (0.5–10 mM for AMP, 0.5–10 mM for ATP) using 3.2 mM as constant saturation concentration. Apparent *K*_M_, *k*_cat_, and *k_cat_/K_M_*, were determined by non-linear regression assuming Michaelis–Menten kinetics. Calculations were carried out using the GraphPad Prism 8 (Motulsky and Christopoulos, 2019).

### Homology Modeling

In order to analyze the structural features of *Zg*HGPRT/AMPK a 3D homology model of the enzyme was built. First, the single domain homology models were built by employing Swiss-Model server (Waterhouse et al., 2018), using the best protein templates of known 3D structures for each domain. Thus, HGPRT from *Leptospira interrogans* (PDB id 4QRI) and AMPK structure from *Geobacillus stearothermophilus* (PDB id 1ZIN) were selected as templates. Then, a complete *Zg*HGPRT/AMPK 3D model was built through assembling the best homology models for both, HGPRT and AMPK domains, using replica-exchange Monte Carlo simulations integrated in Domain Enhanced Modeling server (Zhou et al., 2019). A protein-protein docking using ClusPro (Vajda et al., 2017) was performed to ensure the optimum quality of the dual domain homology model.

Once the quaternary structure of the enzyme was defined, the linker sequence combining both monomers was determined by support vector machine (SVM) implemented in Domain linker pRediction using OPtimal features server (DROP) (Ebina et al., 2011). Flexibility was calculated comparing the atomic fluctuation of the loop with the rest of the structure through molecular dynamic simulation (MD). To this end, *Zg*HGPRT/AMPK model was complexed with Hyp, PRPP and Mg^2+^ in the active site of the HGPRT domain, and with AMP, ATP and Mg^2+^ in the active site of AMPK domain. The complex was then immersed in a box of 10,500 TIP3P water molecules that extended 15 Å away from any solute atom, and 20 Na^+^ ions were added to ensure electrical neutrality. Energy refinement followed by unrestrained MD simulations for 30 ns were carried out using the *pmemd_cuda.SPFP* module and the standard ff14SB force field parameter set in AMBER16 (Case et al., 2016). Finally, the *cpptraj* module (Roe and Cheatham, 2013) in AMBER16 was employed for data processing of the calculated trajectories.

### Analytical Methods

The production of nucleotides was measured quantitatively using an ACE EXCEL (5 μm CN-ES 250 × 4.6 mm) equilibrated with 100% triethyl ammonium acetate at a flow rate of 0.8 mL/min. Retention times for the reference natural compounds (hereafter abbreviated according to the recommendations of the IUPAC-IUB Commission on Biochemical Nomenclature) were as follows: adenine (Ade), 10.4 min; adenosine-5′-monophosphate (AMP), 5.5 min; adenosine-5′-diphosphate (ADP), 4.4 min; adenosine-5′-triphosphate (ATP), 3.8 min; guanine (Gua), 4.8 min; guanosine-5′-monophosphate (GMP), 3.2 min; hypoxanthine (Hyp), 4.5 min; inosine-5′- monophosphate (IMP), 3.0 min; xanthine (Xan), 4.4 min; cytosine (Cyt), 3.9 min; uracil (Ura), 4.1 min; thymine (Thy), 7.2 min; thymidine-5′-monophosphate (TMP), 4.4 min.

## Results And Discussion

### Bioinformatics Analysis of *Zg*HGPRT/AMPK

**FIGURE 3:**
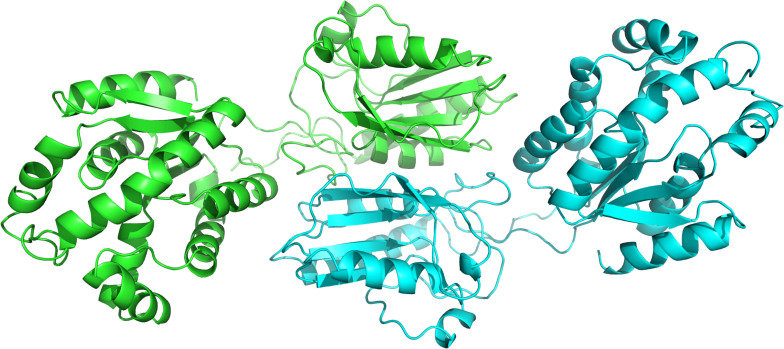
Cartoon representation of the overall *Zg*HGPRT/AMPK model, comprising both subunits (green and blue). The figure was prepared with PyMOL (Delano, 2002).

The genomic information of *Zobellia galactanivorans* has been analyzed and published online (NC_015844.1). After an *in silico* mining, we discovered an ORF that potentially encodes a putative HGPRT/AMPK bifunctional protein annotated throughout the genome. To confirm this, a bioinformatic analysis was performed. The pairwise amino acid sequence alignment using BLASTP^1^ against Protein Data Bank revealed the presence of both canonical, PRT and adenylate kinase domains, encoded in the amino acid sequence (Figure 2). In this sense, with the aim to understand these unusual sequence features, a 3D homology model of *Zg*HGPRT/AMPK was built (Figures 2, 3).

**FIGURE 4:**
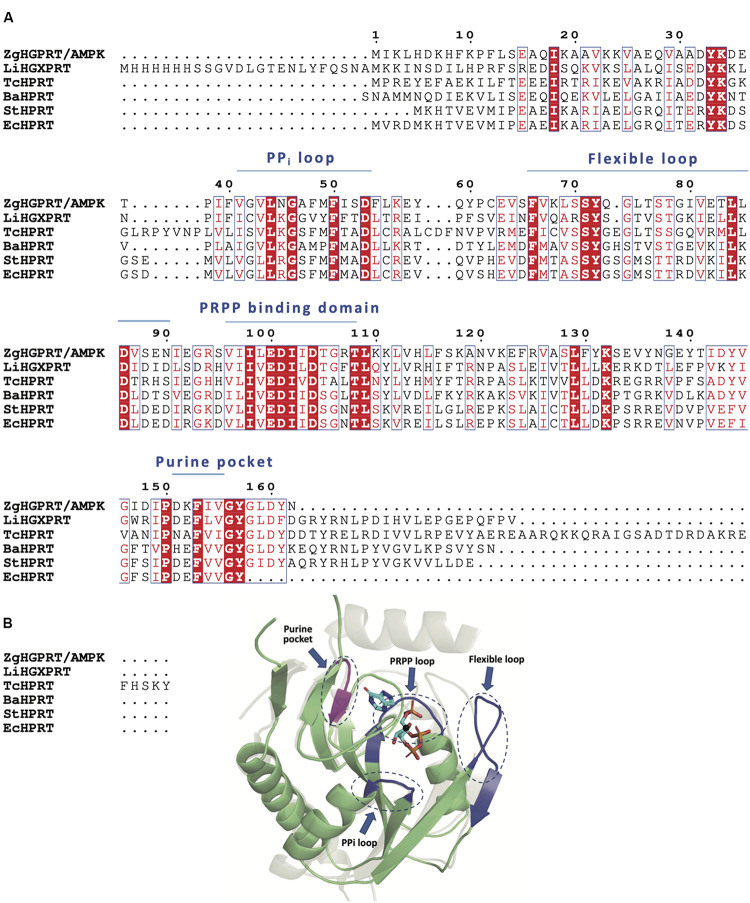
**(A)** Multiple sequence alignment of amino acid sequences of 6-oxopurine PRTs from *Zobellia galactanivorans* (*Zg*HGPRT/AMPK), *Leptospira interrogans* (*Li*HGXPRT, PDB id 4QRI), *Trypanosoma cruzi* (*Tc*HPRT, PDB id 1P19), *Bacillus anthracis* (*Ba*HPRT, PDB id 6D9Q), *Salmonella typhimurium* (*St*HPRT, PDB id 1J7J), *Escherichia coli* (*Ec*HPRT, PDB id 5KNR), **(B)** Overall representation of the active site architecture of *Zg*HGPRT domain based on the structural alignment of *Zg*HGPRT/AMPK homology model (green) with *Trypanosoma cruzi* HRPT (gray) complexed with PRPP and 7-hydroxypyrazolo[4,3-D]pyrimidine (PDB id 1TC2) (sticks), and Mg^2+^ (black sphere). PRPP binding site, PPi and flexible loops (blue) and purine pocket (violet) are encircled with dotted lines. The figure was prepared with PyMOL (Delano, 2002).

According to the multiple sequence alignment of amino acid sequences of closest PRT homologs, *Zg*HGPRT/AMPK would belong to class I PRTs, which display a conserved 13-residue “fingerprint” region (PRPP binding-motif) in their amino acid sequence (Del Arco and Fernández-Lucas, 2017; Figure 4A). Type I PRTs commonly display a core region composed by four- or five-stranded parallel β-sheet surrounded by three α-helices and a hood domain that completes the active site architecture, and is also involved in nucleobase binding (Figure 4B). Some of these catalytic residues are located in several flexible and highly mobile loops which adopt different conformations depending on the binding of substrates or products (Figure 4B). As shown in Figure 4, some essential elements of type I PRTs architecture were found in *Zg*HGPRT/AMPK amino acid sequence such as PPi loop, the flexible loop and PRPP binding domain (including PRPP loop), as well as in the homology model. Finally, the well-known purine pocket of purine PRTs was also found in *Zg*HGPRT/AMPK amino acid sequence.

**FIGURE 5:**
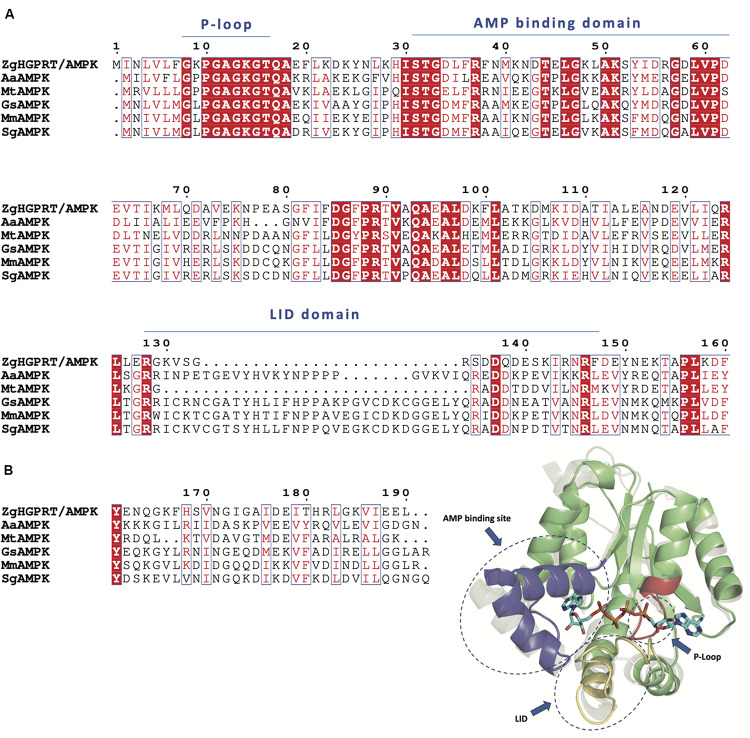
**(A)** Multiple sequence alignment of amino acid sequences of AMPK from *Zobellia galactanivorans* (*Zg*HGPRT/AMPK), *Aquifex aeolicus* (*Aa*AMPK, PDB id 2RGX), *Mycobacterium tuberculosis* (*Mt*AMPK, PDB id 1P4S), *Geobacillus stearothermophilus* (*Gs*AMPK, PDB id 1ZIN), *Marinibacillus marinus* (*Mm*AMPK, PDB id 3FB4), *Sporosarcina globispora* (*Sg*AMPK, PDB id 5X6J). **(B)** Overall representation of the active site architecture of *Zg*AMPK domain based on the structural alignment of *Zg*HGPRT/AMPK homology model with AMPK from *Homo sapiens* (gray) complexed with P1,P4-Bis(5′-adenosyl) tetraphosphate (PDB id 2C95) (sticks). P-Loop site (red), AMP binding site (blue) and LID domain (yellow) are encircled with dotted lines. The core domain (green), including P-loop site and AMPK fingerprint, is also shown. The figure was prepared with PyMOL (Delano, 2002).

Regarding to the AMPK domain, the typical adenylate kinase motifs are present in *Zg*HGPRT/AMPK amino acid sequence (Figure 5A): (i) the P-loop (also known as Walker A motif), that adopts a specific loop shape allowing the accommodation of phosphate moiety from ATP, (ii) the AMP binding domain, which ensures that the adenine group from adenylate is selectively distinguished from other nitrogenous bases, and (iii) the LID domain, which displays high mobility allowing the active site isolation and thus generates a hydrophobic environment, avoiding the hydrolysis and promoting phosphotransferase activity (Formoso et al., 2015; Figure 5A). Moreover, AMPK proteins have a α/β structure formed by 5 β-sheet surrounded by several a-helices (Mukhopadhyay et al., 2010; Figure 5B). Like other AMPKs, *Zg*HGPRT/AMPK shows the typical core domain formed by the P-loop and a high conserved 12-residue AMPK “fingerprint,” including the G-F-P-R sequence that contribute to the protein folding and stabilization (Figure 5B; Mukhopadhyay et al., 2010). As inferred from Figure 5B, ATP would be located between the core and LID domains, while AMP would be sandwiched between the core and the AMP binding site (Figure 5B).

To form the complete monomer of the protein, both HGPRT and AMPK domains are connected by an interdomain linker consisting of 10 amino acids (REVYQLNQKH). This sequence is characterized by charged and polar uncharged amino acids, similar to other linkers from multidomain proteins (Argos, 1990; George and Heringa, 2002). Moreover, this region displays a coil secondary structure (Figure 2) with lower flexibility than the average of the rest of the protein. However, certain degree of flexibility is required to retain the function of individual domains and to allow crucial domain interactions (Reddy Chichili et al., 2013). Nevertheless, Arg, Glu, and Gln residues present in the linker act as rigid spacers to prevent unfavorable interactions between both domains (Chen et al., 2013). Finally, as concluded from the protein-protein docking, multidomain monomers are combined into a dimeric state, which corresponds to the biologically active form of the enzyme (Figure 3).

### Production and Purification of *Zg*HGPRT/AMPK

**FIGURE 6:**
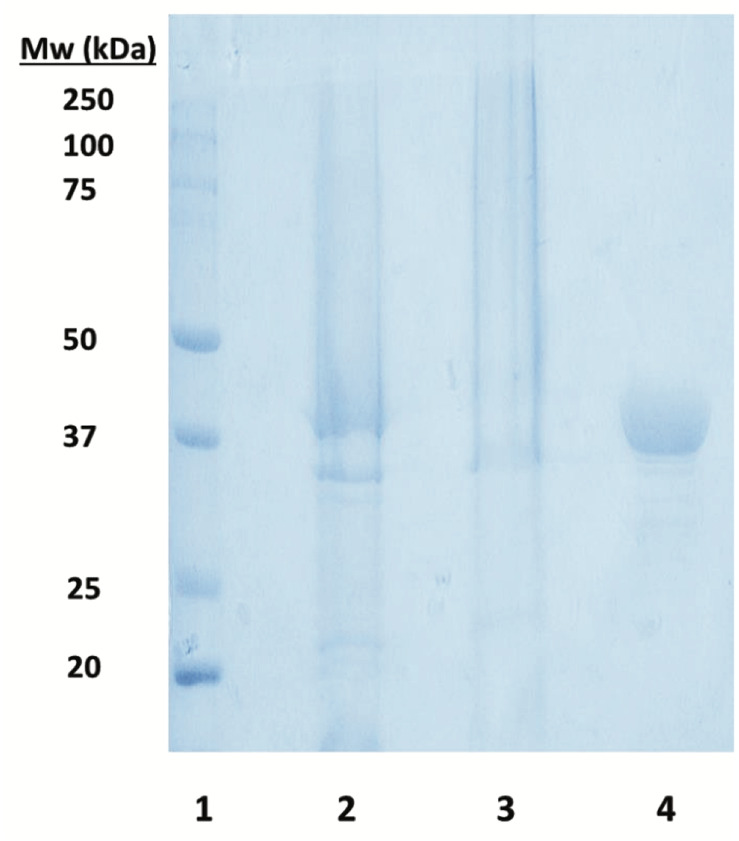
SDS-PAGE analysis of soluble *Zg*HGPRT/AMPK. Lane 1. Precision Plus Protein^TM^ prestained standard from Bio-Rad used as a molecular weight marker. Lane 2. Supernatant obtained after centrifugation of the lysed cells. Lane 3. Pellet obtained after centrifugation of the lysed cells. Lane 4. *Zg*HGPRT/AMPK (15 μg protein) after chromatography purification.

The bioinformatics analysis of *Zobellia galactanivorans* genome (NCBI reference sequence NC_015844.1) displayed the presence of a putative *Zg*HGPRT/AMPK. The putative *hgprt/ampk* gene from *Zobellia galactanivorans* was cloned and overexpressed in *E. coli* BL21(DE3) as described above. The recombinant N-terminal His_6_-tagged *Zg*HGPRT/AMPK was purified by an affinity and an exclusion size chromatographic procedure. SDS-PAGE analysis of the purified enzyme shows only one protein band with an apparent molecular mass around 42 kDa (Figure 6).

The sedimentation velocity experiments revealed *Zg*HGPRT/AMPK as a group of two species with an experimental sedimentation coefficient of 4.51 S (*s*_20,w_ = 4.55 S) (97%) and 7.10 S (*s*_20,w_ = 7.23 S) (3%). The major species found in solution corresponds to a dimer state (Mw = 83.33 kDa) and is compatible with a monomer subunit of 41.65 kDa, a molecular mass similar to that calculated from the amino acid sequence of the His_6_-tagged protein (43.77 kDa). Since *Zg*HGPRT/AMPK is the first dual domain PRT/AMPK protein, there are not previous examples to compare it with. However, different oligomeric states have been described for 6-oxopurine PRTs from several sources, such as dimeric 6-oxo PRTs from *Sulfolobus solfataricus* (*Ss*HGXPRT) and GPRT from *Giardia lamblia* (*Gl*GPRT), tetrameric 6-oxo PRTs from *Thermus thermophilus* (*Tt*HGXPRT and *Tt*XPRT), *Toxoplasma gondii* (*Tg*HGPRT) and *E. coli* (*Ec*XGPRT and *Ec*HPRT), or the hexameric HGPRT from *Pyrococcus horikoshii* (*Pf*HGXPRT) (Del Arco and Fernández-Lucas, 2017). In this respect it seems that *Zg*HGPRT/AMPK could belong to dimeric 6-oxo PRTs.

Regarding to AMPKs, they are generally found in two distinct oligomeric states, the trimeric class commonly present in *Archaea*, and monomeric class more common in *Eubacteria* (Davlieva and Shamoo, 2010).

### Temperature and pH Dependence of *Zg*HGPRT/AMPK Activity

**FIGURE 7:**
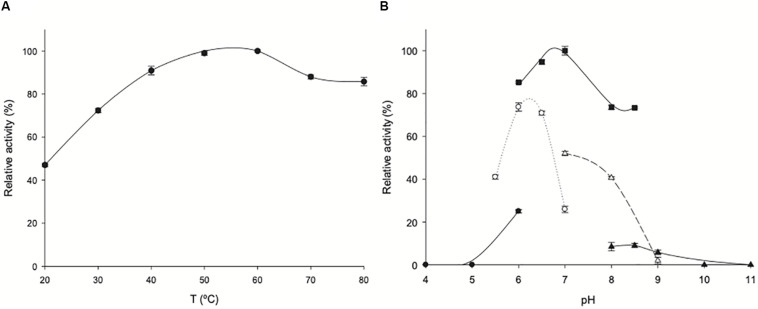
Biochemical characterization of *Zg*HGPRT/AMPK. **(A)** Effect of temperature on *Zg*HGPRT/AMPK activity (•). **(B)** Effect of pH on *Zg*HGPRT/AMPK activity, (•) 50 mM sodium citrate (pH 4–6), (∘) 50 mM MES (pH 5.5–7), (■) 50 mM sodium phosphate (pH 6–8.5), (△) 50 mM Tris–HCl (pH 7–9), (▲) 50 mM sodium borate (pH 8–11). All determinations were carried out in triplicate and the maximum standard deviation value was 2%.

The effect of temperature and pH on *Zg*HGPRT/AMPK activity is shown in Figure 7A. *Zg*HGPRT/AMPK displays high activity (>70%) across a broad temperature range (from 30 to 80°C), with a maximum at 50–60°C, which is higher than those reported for other mesophilic HGPRTs, such as HGXPRT from *Plasmodium falciparum* (*T* = 22°C) (Mbewe et al., 2007), human HGPRT (*T* = 22°C) (Raman et al., 2004) or HPRT from *Trypanosoma cruzi* (*T* = 37°C) (Wenck et al., 2004).

Moreover, the pH profile revealed that *Zg*HGPRT/AMPK displays high activity in a narrow pH range 6–7, with a maximum peak when it was incubated in 50 mM sodium phosphate pH 7 (Figure 7B). In addition, experimental data suggest a strong dependence on the nature of buffer solution for *Zg*HGPRT/AMPK.

### Thermal Stability of *Zg*HGPRT/AMPK

**FIGURE 8:**
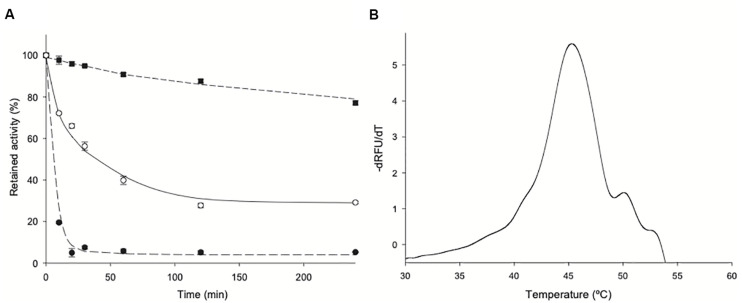
**(A)** Thermal inactivation of *Zg*HGPRT/AMPK at (■) 40°C, (∘) 50°C and (•) 60°C. **(B)** Melting temperature of *Zg*HGPRT/AMPK. All determinations were carried out in triplicate and the maximum standard deviation value was 1.8%.

*Zg*HGPRT/AMPK does not undergo any loss of activity when stored at 4°C in 20 mM sodium phosphate buffer, pH 7 for 365 days. However, a significant loss of activity (≈30%) was observed when stored at −80°C. Furthermore, the effect of temperature on enzyme stability was evaluated by incubating *Zg*HGPRT/AMPK for 4 h in 20 mM sodium phosphate pH 7, in the temperature range 40–60°C (Figure 8A). As expected for a mesophilic enzyme, *Zg*HGPRT/AMPK suffered a high loss of activity when incubated at 50 and 60°C in a brief period of time (relative activity <70% for incubation periods longer than 10 min). In contrast, *Zg*HGPRT/AMPK displayed a high relative activity (around 80%) when stored at 40°C for 240 min. These results agree with thermal denaturation curves, which allowed us to estimate a moderate melting temperature (*T*_m_) of 45.25°C (Figure 8B). As shown in literature, the oligomerization state of the 6-oxopurine PRTs may contribute to the overall stability of the protein and high aggregation states favors the increase of thermal stability in 6-oxo PRTs (Del Arco and Fernández-Lucas, 2017). Considering the dimeric nature of *Zg*HGPRT/AMPK, a high thermal stability was not expected in this case.

### Effect of Divalent Cations on Enzyme Activity

**FIGURE 9:**
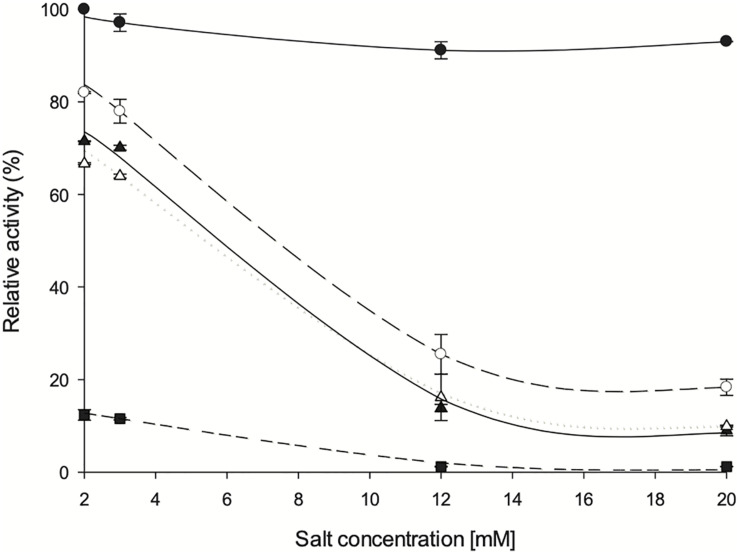
Influence of divalent salts on *Zg*HGPRT/AMPK activity. (•) MgSO_4_, (∘) CoSO_4_, (△) MgSO_4_, (▲) ZnSO_4_ and (■) CaCl_2_. All determinations were carried out in triplicate and the maximum standard deviation value was 1.7%.

Once optimal conditions of pH (7) and temperature (50°C) were stablished, the effect of divalent cations on enzyme activity was assayed (Figure 9). As described for HGPRTs, the presence of two divalent metal ions in the active site of these enzymes is crucial for the catalytic reaction. In fact, they are associated with both the PPi of PRPP and with active site residues (directly or through water molecules). One of these cations is also linked to the purine base through a water molecule, placing the purine substrate for catalysis (Sinha and Smith, 2001).

As expected, an absolute requirement for divalent cations was observed. Thus, the optimum concentration of cations was stablished in a range of 2–3 mM, obtaining best activity values with Mg^2+^ 2 mM. Since high activity values were also observed when using Mn^2+^, it might act as an effective substitute for Mg^2+^, as also described for other HGPRTs such as those from *E. coli*, *Salmonella typhimurium* (Hochstadt, 1978), *Artemia* sp. (Montero and Llorente, 1991) and yeast (Ali and Sloan, 1986). Similar to *Artemia* sp. (Montero and Llorente, 1991) and yeast (Ali and Sloan, 1986), Zn^2+^ can also activate the phosphoribosyl activity on *Zg*HGPRT, although to a lesser extent than Mg^2+^. As shown in Figure 6, high activity values were also obtained with Co^2+^, which is consistent with yeast HGPRT (Ali and Sloan, 1986). Finally, significant low activity values were obtained when using Ca^2+^, so it could not replace Mg^2+^ as observed for HGPRT from *Plasmodium falciparum* (Mbewe et al., 2007). This finding also suggests that Ca^2+^ might act as an inhibitor, as described for APRT from *Artemia* sp. (Montero and Llorente, 1991).

### Effect of Organic Solvents on *Zg*HGPRT/AMPK Activity

**FIGURE 10:**
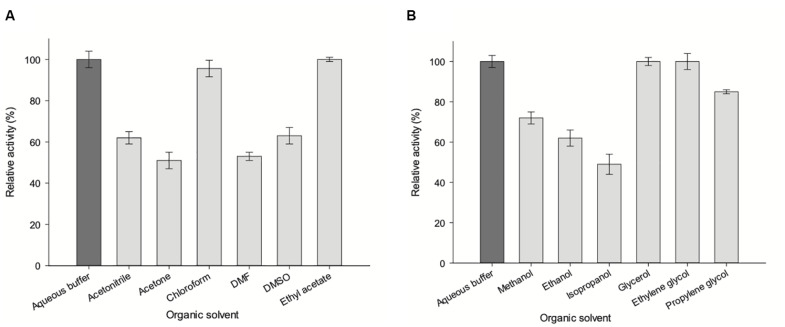
Effect of organic co-solvents (20% v/v) on *Zg*HGPRT/AMPK activity. **(A)** Aprotic polar solvents. **(B)** Alcohols and polyols. All determinations were carried out in triplicate and the maximum standard deviation value was 2%.

To investigate the effect of organic solvents on enzyme activity, phosphoribosyltransferase activity was assayed in the presence of 20% (v/v) polar protic (MeOH, EtOH, isopropanol, glycerol, ethylene glycol, and propylene glycol) and aprotic co-solvents (acetonitrile, acetone, chloroform, N,N-dimethylformamide, DMSO and ethyl acetate) (Fernández-Lucas et al., 2012; Del Arco et al., 2018d). As shown in Figure 10, there is a negligible loss of activity (less than 5%) in presence of 20% glycerol, ethylene glycol, chloroform and ethyl acetate. A moderate loss of activity (less than 40%) was observed in presence of 20% acetonitrile, DMSO, MeOH, EtOH, and propylene glycol. Finally, a significant loss of activity is observed when acetone, DMF or isopropanol were added to the reaction mixture.

A correlation between hydrophobicity and activity (Figure 10A) was observed when using aprotic co-solvents. In this respect, the highest activity values are shown in the presence of 20% of hydrophobic solvents (*log P* > 0), such as ethyl acetate (100% relative activity, *log P* = 0.73) or chloroform (non-miscible solvent, 95.6% relative activity, *log P* = 1.83), whereas a significant activity decrease is observed for hydrophilic solvents (*log P* value < 0), such as acetone (51% relative activity, *log P* = −0.21), acetonitrile (62% relative activity, *log P* = −0.3), DMF (53% relative activity, *log P* = −1.0), DMSO (63% relative activity, *log P* = −1.3) (Table 1). Moreover, we also observe an increase of enzymatic activity when the dielectric constant (*ε*) decreases, displaying the highest activity values for ethyl acetate (*ε* = 6.0) and chloroform (*ε* = 4.8), and the lowest values for DMF (*ε* = 38) and acetone (*ε* = 21) (Table 1).

**TABLE 1:** Effect of 20% of organic co-solvents on *Zg*HGPRT/AMPK activity.

**Solvent**	**Relative activity (%)**	**Log *P***	***ε***
**Aprotic co-solvents**			
Ethyl acetate	100	0.73	6.0
Chloroform (non-miscible)	96	1.83	4.8
Acetone	51	–0.21	21
Acetonitrile	62	–0.3	36.6
DMF	53	–1.0	38.25
DMSO	63	–1.3	47.24
**Protic co-solvents**			
Glycerol	100	–3.03	47
Ethylene glycol	100	–1.80	37
Propylene glycol	85	–0.64	32
EtOH	72	–0.24	25
MeOH	62	–0.76	32.7
Isopropanol	49	0.05	20

*Reaction conditions were 6.5 μg of enzyme in 40 μl at 50°C for 10 min, in presence of 20% organic co-solvents. Substrate concentrations were 2 mM PRPP, 2 mM Hyp, 2.4 mM MgCl_2_ in 50 mM sodium phosphate buffer, pH 7.*

The increase of enzymatic activity in the presence of 20% protic solvents (mono alcohols and polyols) seems to be linked to the increase of dielectric constant (*ε*), in the following order: glycerol (*ε* = 47) > ethylene glycol (*ε* = 37) > propylene glycol (*ε* = 32) ≈ MeOH (*ε* = 32.7) > EtOH (*ε* = 25) > isopropanol (*ε* = 20) (Figure 10B and Table 1). However, higher relative activities were obtained for polyols (100–85%), instead of those obtained for mono alcohols (72–49%), which indicates that the size and/or conformation of polyols also affect the enzyme activity.

### Substrate Specificity

Table 2 summarizes specific activities of *Zg*HGPRT/AMPK for both, phosphoribosyltransferase and adenylate kinase activities. As expected for previous *in silico* analysis, *Zg*HGPRT/AMPK can perform the phosphoribosyltransferase reaction on 6-oxopurines (Hyp and Gua), while neither 6-aminopurines nor pyrimidine bases are substrates for *Zg*HGPRT/AMPK. In addition, no transferase reaction was observed when xanthine was used as acceptor. These results agree with previous reports for purine PRTs since, for the purine salvage, organisms usually display different PRTs, one specific for adenine and one or more responsible for the salvage of 6-oxopurines (Del Arco and Fernández-Lucas, 2017). In this sense, the presence of an *aprt* gene encoding a putative APRT (GenBank: CAZ96936.1) in *Zobellia galactanivorans* genome would support our findings.

**TABLE 2:** Substrate specificity studies for *Zg*HGPRT/AMPK using different nucleotides and nucleobases as substrates.

**Donor**	**Acceptor**	**Product 1**	**Specific activity (U/mg)**	**Product 2**	**Specific activity (U/mg)**
**PRTase activity**					
PRPP	Ade^a^	AMP	n.d.		
	Cyt^a^	CMP	n.d.	−	−
	Gua^b^	GMP	0.47 ± 0.03	−	−
	Hyp^a^	IMP	0.53 ± 0.05	−	−
	Ura^a^	UMP	n.d.	−	−
	Thy^a^	TMP	n.d.	−	−
	Xan^b^	XMP	n.d.	−	−
**Kinase activity**					
ADP	ADP^c^	AMP	0.03 ± 0.004	ATP	0.03 ± 0.004
ATP	AMP^d^	ADP	0.09 ± 0.01	−	−

*^*a*^Reaction conditions were 6.5 μg of enzyme in 40 μl at 50°C for 10 min. Substrate concentrations were 2 mM PRPP, 2 mM base, 2.4 mM MgCl_2_ in 50 mM sodium phosphate buffer, pH 7. ^*b*^Reaction conditions were 6.5 μg of enzyme in 40 μl at 50°C for 10 min. Substrate concentrations were 2 mM PRPP, 2 mM base, 2.4 mM MgCl_2_ in 50 mM Tris–HCl buffer, pH 8. ^*c*^Reaction conditions were 13 μg of enzyme in 40 μl at 50°C for 10–20 min. Substrate concentrations were 6.4 mM ADP, 12 mM MgCl_2_ in 50 mM sodium phosphate buffer, pH 7. ^*d*^Reaction conditions were 13 μg of enzyme in 40 μl at 50°C for 10–20 min. Substrate concentrations were 3.2 mM AMP, 3.2 mM ATP, 12 mM MgCl_2_ in 50 mM sodium phosphate buffer, pH 7.*

**FIGURE 11:**
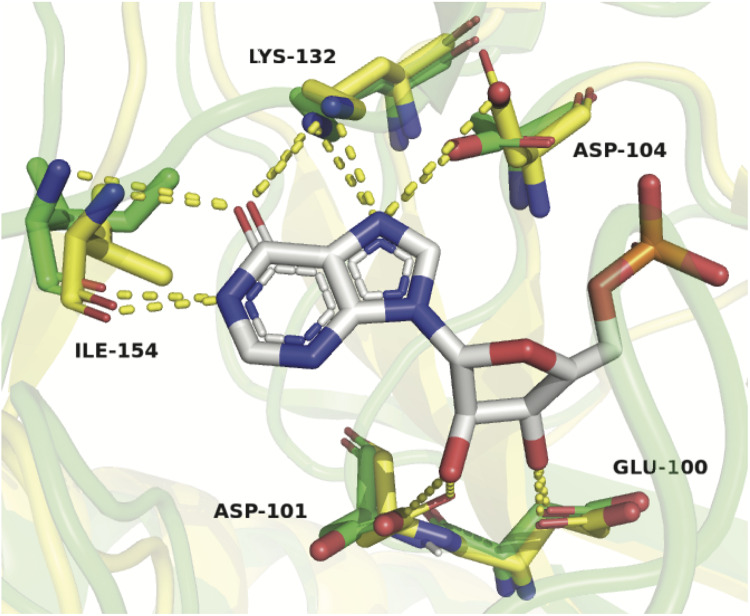
Structural alignment of *Zg*HGPRT/AMPK model (green) and *Tt*HGXPRT (yellow) complexed with IMP (atom-type coloring sticks). Hydrogen bonds formed between the protein residues and IMP are shown as yellow dotted lines. Active site residues in the model are represented by sticks with the atom-type coloring (green). The figure was prepared with PyMOL (Delano, 2002).

To explain these results, a homology model of *Zg*HGPRT/AMPK was built (Figure 3), and then it was superposed with HGXPRT from *Thermus thermophilus* HB8 complexed with IMP (*Tt*HGXPRT, PDB id 3ACD) (Kanagawa et al., 2010; Del Arco et al., 2017). As shown in Figure 11, hypoxanthine binding is stabilized by a network of hydrogen bonds between Asp 104, Lys 132 and Ile 154, and N1, exocyclic O6 and N7 of the purine ring. Due to the absence of any suitable residues for the recognition of exocyclic NH_2_, 6-aminopurines are not properly accommodated for binding and catalysis.

Furthermore, *Zg*HGPRT/AMPK also catalyzed the phosphotransfer reaction between adenine nucleotides. As shown in Table 2, *Zg*HGPRT/AMPK is more active (up to 3 times) when transferring γ-phosphate moiety from ATP to α-phosphate of AMP, than it is for the reverse reaction. In this sense, the reaction equilibrium seems to be shifted toward ADP synthesis. Moreover, the PRTase/kinase activity ratio is around 5-6/1, which indicates a strong preference for Hyp and PRPP instead of adenine nucleotides as substrates. These results might suggest *Zg*HGPRT/AMPK plays a key role in salvage pathway, whereas it is not essential for maintaining the cellular homeostasis of adenine nucleotides.

The presence of the genes in *Zobellia galactanivorans* genome coding other putative enzymes which could also be involved in the metabolism of adenine nucleotides, such as an APRT (GenBank: CAZ96936.1), a ribonucleotide reductase (GenBank: CAZ95963.1), a nucleoside diphosphate kinase (GenBank: CAZ97836.1) and adenylate cyclase (GenBank: CAZ95571.1), would reinforce this hypothesis.

### Kinetic Analysis

Steady-state kinetic studies were conducted at variable concentrations of Hyp or PRPP to determine kinetic parameters (*K_M_, k_cat_*, and *k_cat_/K_M_*). The results are summarized in Table 3. *Zg*HGPRT/AMPK displays a lower *K*_M_ value for Hyp than for PRPP, which agrees with kinetic data described for other type I PRTs (Munagala et al., 1998; Wenck et al., 2004; Kanagawa et al., 2010). Moreover, a lower *k_cat_*, value is observed for Hyp, which is lined with as apparent substrate inhibition at high Hyp concentration (Table 3).

**TABLE 3:** Steady-state kinetic parameters for both IMP and ADP synthesis catalyzed by *Zg*HGPRT/AMPK.

**Parameter**	**Value with variable substrate**			
	**Hyp^a^**	**PRPP^b^**	**ATP^c^**	**AMP^d^**
*K*_M_ (mM)	1.01 ± 0.05	6.77 ± 0.41	0.77 ± 0.04	8.02 ± 0.40
*k*_cat_ (s^–1^)	1.11 ± 0.06	2.82 ± 0.21	0.07 ± 0.003	0.16 ± 0.008
*k_cat_/K_M_*	1.10	0.42	0.10	0.02
*K*_i_ (mM)	11.12 ± 1.02	–	–	–

*^*a*^Concentration range 0.5–17.2 mM Hyp at a fixed PRPP concentration of 5 mM. ^*b*^Concentration range 0.5–17.2 mM PRPP at a fixed Hyp concentration of 5 mM. ^*c*^Concentration range 0.5–10 mM AMP at fixed ATP concentration of 3.2 mM. ^d^Concentration range 0.5–10 mM ATP at fixed AMP concentration of 3.2 mM.*

Regarding to the ADP synthesis, *K*_M_ for AMP is 10-fold higher than *K*_M_ for ATP. This significant difference in the affinity between AMP and ATP is probably due to a more unstable binding of AMP onto AMP binding site, whereas the ATP binding onto LID domain seems to be favored (Zeller and Zacharias, 2015). Moreover, the turnover numbers are in compliance with the release form of products, where ADP liberation from AMP occurs through AMP binding site, and ADP liberation from ATP occurs through CORE domain (Zeller and Zacharias, 2015). Finally, the difference in the catalytic efficiency between AMP and ATP fits the model described by Ådén et al. (2013) where AMP can bind to LID with a very low affinity generating an unproductive complex (Whitford et al., 2007; Ådén et al., 2013; Zeller and Zacharias, 2015).

## Conclusion

Herein we report a novel bifunctional hypoxanthine-guanine phosphoribosyltransferase (HGPRT)/adenylate kinase (AMPK) from *Zobellia galactanivorans* (*Zg*HGPRT/AMPK). *Zg*HGPRT/AMPK is the only two in one HGPRT/AMPK described up to date. Experimental results revealed *Zg*HGPRT/AMPK as a homodimer, active in the pH range 6–7 and in a temperature interval from 30 to 80°C, and also displaying a moderate thermal stability. More interestingly, substrate specificity studies revealed *Zg*HGPRT/AMPK as a bifunctional enzyme, which acts as phosphoribosyltransferase or adenylate kinase depending upon the nature of substrates. Finally, the kinetic analysis of both activities (phosphoribosyltransferase and adenylate kinase), as well as the effect of divalent cations and water-miscible solvents on enzyme activity, were described for a suitable application as biocatalyst for the synthesis of nucleoside-5′-mono, -di and triphosphates.

## Data Availability Statement

All datasets presented in this study are included in the article/supplementary material.

## Author Contributions

JF-L and JA are mainly responsible for the experiment design and for coordinating the research. JA, AH, JB, BH-T, VC-S, and MD contributed to the development and analysis of experimental data. All authors contributed to the article and approved the submitted version.

## Conflict of Interest

The authors declare that the research was conducted in the absence of any commercial or financial relationships that could be construed as a potential conflict of interest.

## Footnotes

1 http://blast.ncbi.nlm.nih.gov/Blast.cgi

## References

[B1] Acosta J. Del Arco J. Martinez-Pascual S. Clemente-Suárez V. Fernández-Lucas J. (2018). One-pot multi-enzymatic production of purine derivatives with application in pharmaceutical and food industry. *Catalysts* 8:9 10.3390/catal8010009

[B2] Ådén J. Weise C. F. Brännström K. Olofsson A. Wolf-Watz M. (2013). Structural topology and activation of an initial adenylate kinase–substrate complex. *Biochemistry* 52 1055–1061. 10.1021/bi301460k 23339454

[B3] Ali L. Z. Sloan D. L. (1986). Activation of hypoxanthine/guanine phosphoribosyltransferase from yeast by divalent zinc and nickel ions. *J. Inorg. Biochem.* 28 407–415. 10.1016/0162-0134(86)80026-5 3546595

[B4] Argos P. (1990). An investigation of oligopeptides linking domains in protein tertiary structures and possible candidates for general gene fusion. *J. Mol. Biol.* 211 943–958. 10.1016/0022-2836(90)90085-Z 2313701

[B5] Brown P. H. Schuck P. (2006). Macromolecular size-and-shape distributions by sedimentation velocity analytical ultracentrifugation. *Biophys. J.* 90 4651–4661. 10.1529/biophysj.106.081372 16565040 PMC1471869

[B6] Case D. Betz R. M. Cerutti D. S. Cheatham T. Darden T. Duke R. (2016). *AMBER 2016.* San Francisco: University of California.

[B7] Chen X. Zaro J. Shen W. C. (2013). “Fusion protein linkers: effects on production, bioactivity, and pharmacokinetics,” in *Fusion Protein Technologies for Biopharmaceuticals: Applications and Challenges*, ed. Schmidt S. R. (Hoboken, NJ: John Wiley & Sons), 57–73. 10.1016/j.addr.2012.09.039

[B8] Davlieva M. Shamoo Y. (2010). Crystal structure of a trimeric archaeal adenylate kinase from the mesophile *Methanococcus maripaludis* with an unusually broad functional range and thermal stability. *Proteins* 78 357–364. 10.1002/prot.22549 19731371

[B9] Del Arco J. Cejudo-Sanches J. Esteban I. Clemente-Suárez V. J. Hormigo D. Perona A. (2017). Enzymatic production of dietary nucleotides from low-soluble purine bases by an efficient, thermostable and alkali-tolerant biocatalyst. *Food Chem.* 237 605–611. 10.1016/j.foodchem.2017.05.136 28764042

[B10] Del Arco J. Fernández-Lucas J. (2017). Purine and pyrimidine phosphoribosyltransferases: a versatile tool for enzymatic synthesis of nucleoside-5’-monophosphates. *Curr. Pharm. Des.* 23 6898–6912. 10.2174/1381612823666171017165707 29046144

[B11] Del Arco J. Fernández-Lucas J. (2018). Purine and pyrimidine salvage pathway in thermophiles: a valuable source of biocatalysts for the industrial production of nucleic acid derivatives. *Appl. Microbiol. Biotechnol.* 102 7805–7820. 10.1007/s00253-018-9242-8 30027492

[B12] Del Arco J. Acosta J. Pereira H. M. Perona A. Lokanath N. K. Kunishima N. (2018a). Enzymatic production of non-natural nucleoside-5’-monophosphates by a *Thermostable uracil* phosphoribosyltransferase. *Chemcatchem* 10 439–448. 10.1002/cctc.201701223

[B13] Del Arco J. Martinez M. Donday M. Clemente-Suarez V. J. Fernández-Lucas J. (2018b). Cloning, expression and biochemical characterization of xanthine and adenine phosphoribosyltransferases from *Thermus thermophilus* HB8. *Biocatal. Biotransform.* 36 216–223. 10.1080/10242422.2017.1313837

[B14] Del Arco J. Martínez-Pascual S. Clemente-Suárez V. J. Corral O. J. Jordaan J. Hormigo D. (2018c). One-pot, one-step production of dietary nucleotides by magnetic biocatalysts. *Catalysts* 8:184 10.3390/catal8050184

[B15] Del Arco J. Sánchez-Murcia P. A. Mancheño J. M. Gago F. Fernández-Lucas J. (2018d). Characterization of an atypical, thermostable, organic solvent-and acid-tolerant 2’-deoxyribosyltransferase from *Chroococcidiopsis thermalis*. *Appl. Microbiol. Biotechnol.* 102 6947–6957. 10.1007/s00253-018-9134-y 29872887

[B16] Del Arco J. Mills A. Gago F. Fernández-Lucas J. (2019a). Structure-guided tuning of a selectivity switch towards ribonucleosides in *Trypanosoma brucei* purine nucleoside 2’-deoxyribosyltransferase. *Chembiochem* 20 2996–3000. 10.1002/cbic.201900397 31264760

[B17] Del Arco J. Pérez E. Naitow H. Matsuura Y. Kunishima N. Fernández-Lucas J. (2019b). Structural and functional characterization of thermostable biocatalysts for the synthesis of 6-aminopurine nucleoside-5’-monophospate analogues. *Bioresour. Technol.* 276 244–252. 10.1016/j.biortech.2018.12.120 30640018

[B18] Delano W. L. (2002). *The PyMOL Molecular Graphics System.* San Carlos, CA: De Lano Scientific.

[B19] Ding Q. Ou L. (2017). NTP regeneration and its application in the biosynthesis of nucleotides and their derivatives. *Curr. Pharm. Des.* 23 6936–6947. 10.2174/1381612823666171024155247 29076413

[B20] Ebina T. Toh H. Kuroda Y. (2011). DROP: an SVM domain linker predictor trained with optimal features selected by random forest. *Bioinformatics* 27 487–494. 10.1093/bioinformatics/btq700 21169376

[B21] el Kouni M. H. (2003). Potential chemotherapeutic targets in the purine metabolism of parasites. *Pharmacol. Ther.* 99, 283–309. 10.1016/S0163-7258(03)00071-8 12951162

[B22] Fernández-Lucas J. (2015). Multienzymatic synthesis of nucleic acid derivatives: a general perspective. *Appl. Microbiol. Biotechnol.* 99 4615–4627. 10.1007/s00253-015-6642-x 25952113

[B23] Fernández-Lucas J. Acebal C. Sinisterra J. V. Arroyo M. de la Mata I. (2010). Lactobacillus reuteri 2’-deoxyribosyltransferase, a novel biocatalyst for tailoring of nucleosides. *Appl. Environ. Microbiol.* 76 1462–1470. 10.1128/aem.01685-09 20048065 PMC2832402

[B24] Fernández-Lucas J. Fresco-Taboada A. de la Mata I. Arroyo M. (2012). One-step enzymatic synthesis of nucleosides from low water-soluble purine bases in non-conventional media. *Bioresour. Technol.* 115 63–69. 10.1016/j.biortech.2011.11.127 22197334

[B25] Formoso E. Limongelli V. Parrinello M. (2015). Energetics and structural characterization of the large-scale functional motion of adenylate kinase. *Sci. Rep.* 5:8425. 10.1038/srep08425 25672826 PMC4325324

[B26] Fresco-Taboada A. de la Mata I. Arroyo M. Fernández-Lucas J. (2013). New insights on nucleoside 2’-deoxyribosyltransferases: a versatile biocatalyst for one-pot one-step synthesis of nucleoside analogs. *Appl. Microbiol. Biotechnol.* 97 3773–3785. 10.1007/s00253-013-4816-y 23529679

[B27] George R. A. Heringa J. (2002). An analysis of protein domain linkers: their classification and role in protein folding. *Protein Eng. Des. Sel.* 15 871–879. 10.1093/protein/15.11.871 12538906

[B28] Hochstadt J. (1978). Hypoxanthine phosphoribosyltransferase and guanine phosphoribosyltransferase from enteric bacteria. *Methods Enzymol.* 51 549–558. 10.1016/S0076-6879(78)51077-X 692401

[B29] Kamel S. Yehia H. Neubauer P. Wagner A. (2019). “Enzymatic synthesis of nucleoside analogues by nucleoside phosphorylases,” in *Enzymatic and Chemical Synthesis of Nucleic Acid Derivatives*, ed. Fernández-Lucas M. J. (Weinheim: Wiley-VCH), 1–28. 10.1002/9783527812103.ch1

[B30] Kanagawa M. Baba S. Ebihara A. Shinkai A. Hirotsu K. Mega R. (2010). Structures of hypoxanthine-guanine phosphoribosyltransferase (TTHA0220) from *Thermus thermophilus* HB8. *Acta Crystallogr. Sect. F Struct. Biol. Cryst. Commun.* 66 893–898. 10.1107/S1744309110023079 20693661 PMC2917284

[B31] Lapponi M. J. Rivero C. W. Zinni M. A. Britos C. N. Trelles J. A. (2016). New developments in nucleoside analogues biosynthesis: a review. *J. Mol. Catal. B Enzym.* 133 218–233. 10.1016/j.molcatb.2016.08.015

[B32] Laue T. M. Shah B. D. Ridgeway T. M. Pelletier S. L. (1992). “Computer aided interpretation of analytical sedimentation data for proteins,” in *Analytical Ultracentrifugation In Biochemistry And Polymer Science*, eds Harding S. E. Horton J. C. Rowe A. J. (Cambridge: Royal Society of Chemistry), 90–125.

[B33] Lewkowicz E. S. Iribarren A. M. (2017). Whole cell biocatalysts for the preparation of nucleosides and their derivatives. *Curr. Pharm. Design.* 23 6851–6878. 10.2174/1381612823666171011101133 29022510

[B34] Mbewe B. Chibale K. McIntosh D. B. (2007). Purification of human malaria parasite hypoxanthine guanine xanthine phosphoribosyltransferase (HGXPRT) using immobilized reactive red 120. *Protein Expr. Purif.* 52 153–158. 10.1016/j.pep.2006.09.014 17097304

[B35] Mikhailopulo I. A. (2007). Biotechnology of nucleic acid constituents-State of the art and perspectives. *Curr. Org. Chem.* 11 317–335. 10.2174/138527207780059330

[B36] Minton A. P. (1997). Alternative strategies for the characterization of associations in multicomponent solutions via measurement of sedimentation equilibrium. *Prog. Colloid Polym. Sci.* 107 11–19. 10.1007/BFb0118010

[B37] Montero C. Llorente P. (1991). Artemia purine phosphoribosyltransferases. Purification and characterization. *Biochem. J.* 275 327–334. 10.1042/bj2750327 1850982 PMC1150056

[B38] Motulsky H. Christopoulos A. (2019). *Fitting Models to Biological Data using Linear and Nonlinear Regression. A Practical Guide to Curve Fitting.* New York, NY: Oxford University Press.

[B39] Mukhopadhyay A. Kladova A. V. Bursakov S. A. Gavel O. Y. Calvete J. J. Shnyrov V. L. (2010). Crystal structure of the zinc-, cobalt-, and iron-containing adenylate kinase from *Desulfovibrio gigas*: a novel metal-containing adenylate kinase from Gram-negative bacteria. *J. Biol. Inorg. Chem.* 16 51–61. 10.1007/s00775-010-0700-8 20821240

[B40] Munagala N. R. Chin M. S. Wang C. C. (1998). Steady-state kinetics of the hypoxanthine-guanine-xanthine phosphoribosyltransferase from *Tritrichomonas foetus*: the role of threonine-47. *Biochemistry* 37 4045–4051. 10.1021/bi972515h 9521725

[B41] Niesen F. H. Berglund H. Vedadi M. (2007). The use of differential scanning fluorimetry to detect ligand interactions that promote protein stability. *Nat. Protoc.* 2 2212–2221. 10.1038/nprot.2007.321 17853878

[B42] Panayiotou C. Solaroli N. Karlsson A. (2014). The many isoforms of human adenylate kinases. *Int. J. Biochem. Cell. B* 49 75–83. 10.1016/j.biocel.2014.01.014 24495878

[B43] Pérez E. Sánchez-Murcia P. A. Jordaan J. Blanco M. D. Mancheño J. M. Gago F. (2018). Enzymatic synthesis of therapeutic nucleosides using a highly versatile purine nucleoside 2’-deoxyribosyltransferase from *Trypanosoma brucei*. *Chemcatchem* 10 4406–4416. 10.1002/cctc.201800775

[B44] Raman J. Sumathy K. Anand R. P. Balaram H. (2004). A non-active site mutation in human hypoxanthine guanine phosphoribosyltransferase expands substrate specificity. *Arch. Biochem. Biophys.* 427 116–122. 10.1016/j.abb.2004.04.014 15178494

[B45] Reddy Chichili V. P. Kumar V. Sivaraman J. (2013). Linkers in the structural biology of protein–protein interactions. *Protein Sci.* 22 153–167. 10.1002/pro.2206 23225024 PMC3588912

[B46] Roe D. R. Cheatham T. E. I. I. I. (2013). PTRAJ and CPPTRAJ: software for processing and analysis of molecular dynamics trajectory data. *J. Chem. Theory Comput.* 9 3084–3095. 10.1021/ct400341p 26583988

[B47] Serra I. Ubiali D. Piškur J. Munch-Petersen B. Bavaro T. Terreni M. (2017). Immobilization of deoxyadenosine kinase from *Dictyostelium discoideum* (DddAK) and its application in the 5’-phosphorylation of arabinosyladenine and arabinosyl-2-fluoroadenine. *Chem. Select* 2 5403–5408. 10.1002/slct.201700558

[B48] Sinha S. C. Smith J. L. (2001). The PRT protein family. *Curr. Opin. Struct. Biol.* 11 733–739. 10.1016/S0959-440X(01)00274-3 11751055

[B49] Ubiali D. Speranza G. (2019). “Enzymatic phosphorylation of nucleosides,” in *Enzymatic and Chemical Synthesis of Nucleic Acid Derivatives, in Enzymatic and Chemical Synthesis of Nucleic Acid Derivatives*, eds Fernández-Lucas J. Camarasa M. J. (Weinheim: Wiley), 29–42. 10.1002/9783527812103.ch2

[B50] Vajda S. Yueh C. Beglov D. Bohnuud T. Mottarella S. E. Xia B. (2017). New additions to the ClusPro server motivated by CAPRI. *Proteins* 85 435–444. 10.1002/prot.25219 27936493 PMC5313348

[B51] Waterhouse A. Bertoni M. Bienert S. Studer G. Tauriello G. Gumienny R. (2018). SWISS-MODEL: homology modelling of protein structures and complexes. *Nucleic Acids Res.* 46 W296–W303. 10.1093/nar/gky427 29788355 PMC6030848

[B52] Wenck M. A. Medrano F. J. Eakin A. E. Craig S. P. (2004). Steady-state kinetics of the hypoxanthine phosphoribosyltransferase from *Trypanosoma cruzi*. *BBA Proteins Proteom.* 1700 11–18. 10.1016/j.bbapap.2004.03.009 15210120

[B53] Whitford P. C. Gosavi S. Onuchic J. N. (2007). Conformational transitions in adenylate kinase. *J. Biol. Chem.* 283 2042–2048. 10.1074/jbc.m707632200 17998210

[B54] Yoshikawa M. Kato T. Takenishi T. (1967). A novel method for phosphorylation of nucleosides to 5’-nucleotides. *Tetrahed. Lett.* 8 5065–5068. 10.1016/S0040-4039(01)89915-9 6081184

[B55] Yoshikawa M. Kato T. Takenishi T. (1969). Studies of phosphorylation. III. Selective phosphorylation of unprotected nucleosides. *Bull. Chem. Soc. Jpn.* 42 3505–3508. 10.1246/bcsj.42.3505 27682988

[B56] Zeller F. Zacharias M. (2015). Substrate binding specifically modulates domain arrangements in adenylate kinase. *Biophys. J.* 109 1978–1985. 10.1016/j.bpj.2015.08.049 26536274 PMC4643206

[B57] Zhou X. Hu J. Zhang C. Zhang G. Zhang Y. (2019). Assembling multidomain protein structures through analogous global structural alignments. *PNAS* 116 15930–15938. 10.1073/pnas.1905068116 31341084 PMC6689945

